# Cross-species gene normalization by species inference

**DOI:** 10.1186/1471-2105-12-S8-S5

**Published:** 2011-10-03

**Authors:** Chih-Hsuan Wei, Hung-Yu Kao

**Affiliations:** 1 Department of Computer Science and Information Engineering, National Cheng Kung University, Tainan, Taiwan, R.O.C

## Abstract

**Background:**

To access and utilize the rich information contained in the biomedical literature, the ability to recognize and normalize gene mentions referenced in the literature is crucial. In this paper, we focus on improvements to the accuracy of gene normalization in cases where species information is not provided. Gene names are often ambiguous, in that they can refer to the genes of many species. Therefore, gene normalization is a difficult challenge.

**Methods:**

We define “gene normalization” as a series of tasks involving several issues, including gene name recognition, species assignation and species-specific gene normalization. We propose an integrated method, GenNorm, consisting of three modules to handle the issues of this task. Every issue can affect overall performance, though the most important is species assignation. Clearly, correct identification of the species can decrease the ambiguity of orthologous genes.

**Results:**

In experiments, the proposed model attained the top-1 threshold average precision (TAP-k) scores of 0.3297 (k=5), 0.3538 (k=10), and 0.3535 (k=20) when tested against 50 articles that had been selected for their difficulty and the most divergent results from pooled team submissions. In the silver-standard-507 evaluation, our TAP-k scores are 0.4591 for k=5, 10, and 20 and were ranked 2^nd^, 2^nd^, and 3^rd^ respectively.

**Availability:**

A web service and input, output formats of GenNorm are available at http://ikmbio.csie.ncku.edu.tw/GN/.

## Background

In recent years, the amount of biological literature has increased rapidly. Text-mining techniques for extracting information from this work are not completely reliable [1]. Extracting information on proteins automatically and precisely is very important and difficult. Many methods have been developed, and they mainly consist of two tasks. Relation extraction identifies the relationships among biomedical entities in the literature. In extracting relations, each biomedical entity, such as a gene, protein or disease, in an article is mapped to its database identifier. This task is called name entity normalization. This is particularly challenging given the high ambiguity in biology and biomedicine of many entity names, such as the gene/protein name [2-7], species name [8-10], and the chemical/compound name [11,12].

The Critical Assessment of Information Extraction Systems in Biology (BioCreative), a renowned competition in the field of biological text mining, covers a variety of important issues. BioCreative III addressed three text-mining tasks in the domain of molecular biology: gene normalization (GN), protein-protein interactions (PPI), and an interactive demonstration task for gene indexing and retrieval (IAT). The GN task in BioCreative III was similar to the same tasks in previous BioCreative competitions [4-6], in that the goal was to map genes or proteins mentioned in the literature to standard database identifiers. The GN tasks of BioCreative II.5 & III were more difficult than those in preceding challenges. In particular, species information was not provided, and the normalization targets were changed to full-text articles. The GN tasks of BioCreative I & II had been limited to a specific species, such as a human, fly, yeast, mice, and the tasks extracted information from abstracts in the literature. These two differences resulted in a more challenging evaluation than the previous competitions.

Many gene normalization studies have focused on GN tasks in which species information is provided. Hakenberg [13] developed an outstanding gene-name normalization system, winning the BioCreative II name entity normalization task. ProMiner [3] is a well-known gene-name normalization system that employs a dictionary-based approach and relies on manual curation. GENO [7] is a high-performing and efficient gene-name normalization system. It applies the TF-IDF weighting scheme and calculates semantic similarity scores to resolve ambiguous terms. All of these systems perform well, obtaining F-measures of 80%. Later, Hakenberg [2] developed a cross-species normalization system, GNAT, which considers 13 different species and obtains an F-measure of 81.4%.

The interactor normalization task (INT) of BioCreative II.5 did not provide species information and focused on full-text articles. Owing to the two difficult characteristics of this task, the normalization results might seem surprisingly low [5]. Hakenberg et al [14] modified their previous work, including GNAT and a gene mention recognition system (BANNER), and obtained the highest precision (F-measure 43.4%). They disambiguated species and assigned candidate identifiers to proteins mentions. Chen et al. [15] developed a Biological Literature Miner (BioLMiner) system to handle the INT and IPT(interaction pair task) tasks. Two of their subsystems involved in the INT task are the gene mention recognizer (GMRer) and a gene normalizer (GNer). These two subsystems were developed based on support vector machine (SVM) and a conditional random field with designed informative features. Verspoor et al. [16] introduced an approach using fuzzy dictionary lookup to detect mentions of proteins. They also described several strategies for disambiguating species associated with gene mentions; these strategies operated globally (throughout the document) and locally (in the immediate vicinity of a protein mention). Sætre et al. [17] used many subcomponents to produce the AkaneRE system, which obtained the highest recall (68.3%). The AkaneRE is provided by the U-Compare system, and it includes sentence boundary detection, tokenization, stemming, part-of-speech tagging, parsing, named-entity recognition, generation of potential relations, generation of features for each relation, and finally, assignment of confidence scores and ranking of candidate relations. Dai et al. [18] used a three-stage normalization algorithm with a ranking method to handle this task. For interactor ranking, candidate identifiers are ranked by SVM classifier with baseline features and template features.

The purpose of the GN task in BioCreative III is to produce a list of the EntrezGene [19] identifiers of all species for gene mentions in full-text articles. This task is a complicated challenge involving three issues: **gene-mention variation**, **orthologous gene ambiguity** and **intra-species gene ambiguity**. Gene-mention variation occurs when a gene in a dictionary has multiple names. Gene names in the literature also show high variation, including orthographical variation (e.g., “TLR7” and “TLR-7”), morphological variation (e.g., “GHF-1 transcriptional factor” and “GHF-1 transcription factor”), syntactic variation, variation with abbreviations, and variation in enumeration (e.g., “TLR7/8” and “TLR7, TLR8”) [20,21]. Orthologous genes usually belong to several species. To solve the GN task accurately, gene mentions must be assigned to the correct species and normalized to their own database identifiers. This is the most complicated step, and it arises from the extreme ambiguity of orthologous genes. For example, the ESR1 gene is associated with 22 Entrez Gene Ids, which belong to several different species. It is very difficult to normalize gene identifiers that lack species information. Intra-species gene ambiguity can occur when different genes have the same name. For example, the name “CAS” may refer to multiple distinct identifiers, such as Entrez Gene Id:1434 (“Cellular apoptosis susceptibility protein”) or Entrez Gene Id:9564 (“Breast cancer anti-estrogen resistance 1”).

In this study, we propose an integrative method, GenNorm, to handle the three issues of the GN task. Our approach uses three modules, the gene name recognition (GNR) module, the species assignation (SA) module and the species-specific gene normalization (SGN) module. Good GNR processing can insure a high quality of gene mentions and reduce gene mention variation. SA is critical. Given a gene mention, it is essential to know to which species the gene belongs. Species information can help identify orthologous genes and help resolve the species discussed in each article. Ignoring the SA can lead to severe problems with gene ambiguity. SGN is the last and significant for GN; this module is responsible for intra-species gene ambiguity.

## Methods

**Figure 1:**
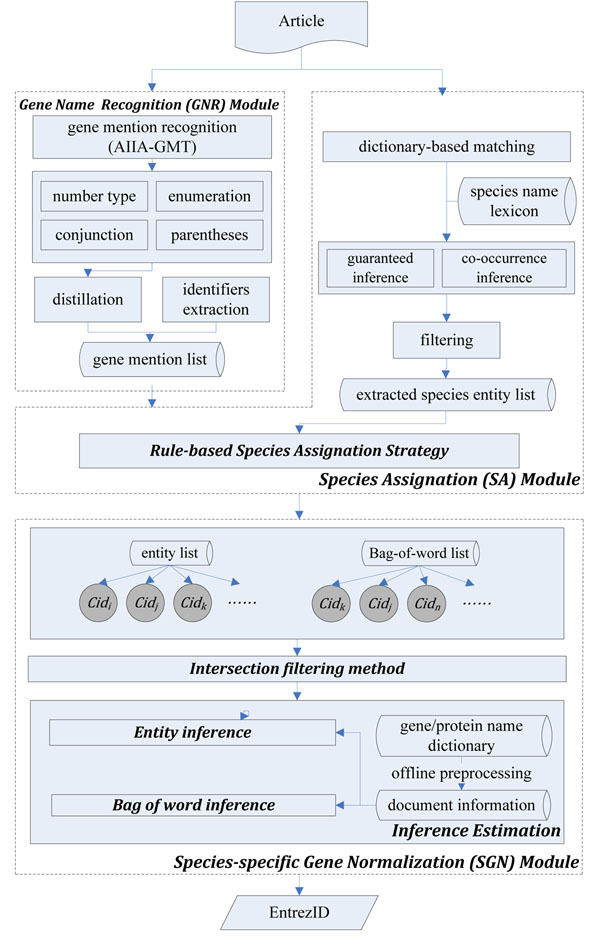
**Architecture of GenNorm.** GenNorm includes gene name recognition (GNR), species assignation (SA) and species-specific gene name normalization modules.

For the GN task, we developed an integration method consisting of three modules, as shown in Figure 1. The GNR module extracts gene mentions from full-text articles. This module applies a well-known system for tagging gene mentions to tokenize them by proposed post-processing rules. The distillation strategy then filters non-gene names. The SA module applies a dictionary-based matching method with two robust inferring strategies to generate the species entity list. The species lexicon combines species and cell names (which can indicate their own species) to cover all kinds of species mentions. Then, the species assignation strategy uses contextual information to decrease the ambiguity of each gene mention. Finally, the SGN module uses an inference network model to handle intra-species gene ambiguity and gene-name variation.

### Gene name recognition module

Our system uses the name-entity tagging tool AIIA-GMT [22]. This tool is an XML-RPC client of a web server that recognizes named entities in biomedical articles. A general entity recognition process cannot collect sufficient information for entity normalization. These systems usually treat lightly or ignore informal, simplified naming descriptions, such as abbreviated names, enumeration mention descriptions, and names with conjunctions. Thus, we propose a post-processing step which can enhance the ability of general-purpose recognition systems.

Due to the varied naming styles of gene names in the biomedical literature, a tagged entity will not always exactly match a gene name in the dictionary. To address this issue, four translation rules of the post-processing step, i.e., the “number type”, “conjunctions”, “enumerations”, and “parentheses”, are applied to tokenize gene names (Table 1).

**Table 1:** Several steps of post-processing of the GNR module.

Step	Rules	Example
Number type	Roman → Arabic Greek → English	I, II, III Roman→ “1, 2, 3” Arabic alpha, beta, gamma → “a”, ”b”, ”g”
Conjunctions	Split entity by and/or	GABARAP and light chain 3→“GABARAP” & “light chain 3” furin or proprotein convertase → “furin” and “proprotein convertase”
Enumerations	Enumeration→ gene mentions	Robo 1/2 → “Robo 1” and “Robo 2” SMADs 1, 5 and 8 → “SMAD 1” and “SMAD 5” and “SMAD 8”
Parentheses	Split entity by parentheses	fibroblast growth factor-2 (FGF-2)-interacting-factor → “FGF-2” and “fibroblast growth factor 2 interacting factor” gamma carboxyglutamic acid (Gla) → “gamma carboxyglutamic acid” and “Gla”

The first rule is the number type. Numbers of different subunit type, e.g., Roman, Arabic and Latin, are unified. Second, entities with conjunctions are split. Sometimes, two or more gene names have been combined into one mention by several conjunctions. This mention is split into several mentions. In the enumeration step, we extract the Arabic and Roman types by splitting conjunctions and combining the mutual family name with each Arabic or Roman type. At this step, an enumeration entity with the sequential numbers represents several gene names belonging to the same family. The entity is separated into several different gene names sharing their mutual family name. In the last rule, abbreviations in the parentheses of a gene entity are isolated. The “protein(s)” or “gene(s)” are then removed from the mentions, e.g., “MURF 3 protein” becomes “MURF 3”.

After the post-processing, the method applies a distillation strategy to prevent false tagging. This step focuses on protein families, group and complex names, and non-gene terms from the tagged entities. In our performance observation in training data, filtering the protein families, group and complex names improved the precision, but the recall was lost. Therefore, the F-measure did not change, but the TAP-K score was improved. Thus, these equivocal terms are filtered in this strategy.

We apply four regular expressions (Table 2) to remove the filtering targets, which might include family names, figure names, or antibodies, for example. Some gene identifiers are mentioned in the articles, and these identifiers can be directly matched to their own species; however, most general taggers of gene mentions cannot extract identifiers mentions. For this particular situation, we attach an “identifier extraction” to the GNR module. To collect identifier mentions, we combine several kinds of gene identifiers, such as swissprot_id and SGD_id, from the locusTaganddbXrefs attributes of the **gene_info** table of the EntrezGene database and use the combined corpus to match identifier mentions. To reduce the computational cost, tokens with Arabic numerals or alphabetic characters are extracted from the articles. The extraction of candidate mention of identifiers is handled by two regular expressions: /(\S*[0-9]+\S*[A-Za-z]+\S*)([^0-9A-Za-z]+.*)$/ and /(\S*[A-Za-z]+\S*[0-9]+\S*)([^0-9A-Za-z]+.*)$/. The first matching by pair of parentheses is used to match the target tokens. The second pair of parentheses extracts the part of sentences after the matched target tokens. It is because that one sentence may contain more than one target token. Particular symbols, including “white”, “hyphen”, “dot” and “underline”, are then removed from the tokens and gene identifiers before matching, e.g., “YCL057C-A” becomes “YCL057CA”. After this adjustment, the tokens are compared with the gene identifiers. Tokens that exactly match gene identifiers are possible Entrez Gene Ids.

**Table 2:** Regular expressions used in the distillation strategy

Filter set	Regular expression
Family name	/([A-Za-z0-9]+)[^A-Za-z0-9](cell|family|subfamily|superfamily|domain|promotor)/
Attachment	/(fig|figure|video|movie|tab|table)[^A-Za-z0-9]+([A-Za-z0-9]+)/
Antibody name	/(anti)[^A-Za-z0-9]+([A-Za-z0-9]+)[^A-Za-z0-9]+(antibody|antibodies)/
Biomedical words	/([A-Za-z0-9]+)[^A-Za-z0-9]+(binding peptide|pathway|domain|cell|function|isoforms)/

Lastly, the extracted entities are dumped into a “bag of words,” as determined by their punctuation, symbols, and spaces. For example, “Hypoxia-inducible factor-1 alpha” is split into “Hypoxia“, “inducible”, “factor”, “1” and “alpha”, each of which is stored in the bag-of-words list.

### Species assignation module

To assign a suitable species to each gene mention, the first step is to extract species mentions from articles. We propose two robust inference strategies combined with a dictionary-based matching method. The species name collection aggregates three different species name lexicons: the NCBI taxonomy, list of cell lines from Wikipedia and the corpus of Linnaeus [9]. Sometimes, species do not map to any Entrez Gene Ids. For example, *Escherichia* of tax_id:561 does not map to any Entrez Gene Id. Such species are removed. The number of species in the original lexicons is 570,679; after filtering, the number species in the combined lexicon is 6,764.

Synonyms of every species in the lexicon are used to detect species names using dictionary-based matching. A species may have a variety of abbreviation names, e.g., “Escherichia coli strain k-12” is same as “E.coli k-12” and “E. coli k-12”. Some species have an especially large number of synonyms. To handle this case, a dictionary extension strategy is used. We automatically generate additional synonyms from each species name by replacing the genus name with its first letter and a dot and potentially a white space [10], e.g., “E. coli”. These species synonyms are then added if they do not already occur in the collection of lexicons. All uppercase letters are then changed to a lowercase form. For example, Taxonomy ID (tax_id): 83333 contains several synonyms, including “escherichia coli k-12”, “escherichia coli k12”, “e.coli k-12” and “e. coli k-12”.

Checking all synonyms of a species is a time-consuming task. To enhance the computation speed, all synonymous names of a species are integrated and transformed into a regular expression automatically. In other words, the regular expression of a tax_id contains all existing names of the species. For the given example, tax_id:83333 would lead to the expression “e(?:\. ?coli k\-12|scherichia coli k\-?12)”.

The NCBI Taxonomy is a hierarchical structure of species types. Our collection only includes four types, “species”, “no rank”, “subspecies” and “variants”. Additionally, we add the genus, taken from the first word of each species’ scientific name, to the species dictionary. These genus names will be used to infer the correct tax_id, as will be discussed below. (We set the priorities for the disambiguation of species names as “species” > “no rank” > “subspecies” > “variants” > “genus name”, with the species type on the left a higher priority than the type on the right). When two species have the same name, the lower priority one is eliminated.

There are some additional cases of non-matching results. First, some species entities are genus names. These entities in the same article always indicate a mutual species of the genus, e.g., “Arabidopsis” is recognized as “Arabidopsis thaliana” when the same article includes both entities. Second, family species will share the same species name. An example is shown in Table 3 for the ambiguous *Escherichia coli* species family, which includes 45 taxonomy identifiers, such as 511145, 431946, and 511693. For tax_id:511145, the last subtype of the species is “mg1655”, and it can indicate the species mention “Escherichia coli” to tax_id:511145.

**Table 3:** Species family of Escherichia coli (tax_id:562) including 45 species.

tax id	Species scientific name
199310	escherichia coli cft073
316385	escherichia coli str. k-12 substr. dh10b
316401	escherichia coli etec h10407
331111	escherichia coli e24377a
331112	escherichia coli hs
344610	escherichia coli 53638
413997	escherichia coli b str. rel606
431946	escherichia coli se15
83333	escherichia coli k-12
511145	escherichia coli str. k-12 substr. mg1655
…	…

**Figure 2:**
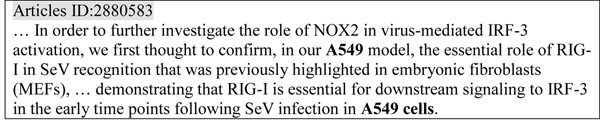
**Example of guaranteed inference.** The unguaranteed species name-”A549” can be inferred to human species by guaranteed species name-“A549 cells”.

To handle these cases, we devised two robust strategies of robust inference. (1) Guaranteed inference: guaranteed entities can be used to disambiguate unguaranteed entities*.* The complete species name is guaranteed to indicate tax_id, but genus names, ambiguous cell names and abbreviations are unguaranteed. The substitution of genus for species name can also be described as a type of anaphora. Unguaranteed entities always occur with the guaranteed name in articles, e.g., “Arabidopsis” accompanies “Arabidopsis thaliana” and “A549” accompanies “A549 cell(s)”. In reverse, “A549” cannot imply “A549 cell” when the article does not contain the complete species name “A549 cell(s)”. The guaranteed entity can imply the unguaranteed entities that indicate the same species. As an example (Figure 2), “A549 cells” can imply that “A549” indicates an A549 cell. Thus, the “A549 cell” mention frequency is two in this paragraph. (2) Co-occurrence inference: the species sub-type can disambiguate the species name and genus name, when the sub-type appears after the species and genus names in the same sentence. The sub-types in the NCBI Taxonomy include strain, substrain, variant, subspecies, pathovars and biovar. For example, “MG1655” is a substrain of “E. coli K12”.

**Figure 3:**
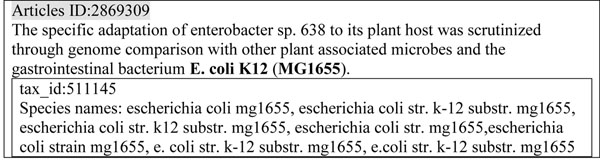
**Example of co-occurrence inference.** The substrain-“MG1655” can infer the species name-“E. coli K12” to “E. coli str. k-12 substr. mg1655” (tax_id:511145).

Figure 3 shows how “E. coli K12 (MG1655)” appears different from all synonyms of tax_id:511145. Dictionary-based matching is not useful in this situation. In our observation, each subtype can be unambiguously assigned to one species. Thus, the MG1655 can disambiguate “Escherichia”, “E. coli” and “E. coli K12” to the specific species (tax_id:511145).

We show in Table 4 the types of species mentions by species extracting step in species assignation module. The targets of guaranteed inference are “genus name” and “cell line”, and the target of co-occurrence inference is “sub-type”. The total percentage of inference targets among species mentions is 11.3% (8.06%+2.04%+1.20%). However, these targets are not a major part of species mentions. It is nonetheless the most difficult aspect of detecting species mentions. Lastly, a set of high-false-positive aggregated from species mentions is shown in Table 5. The first row is collected by Linnaeus software [9]. Candidate species terms matching high false positive terms are removed. Two experiment descriptors in the second row contain “yeast”; similar species names would lead to false positives, and the “yeast” in these two experiment terms is removed. In addition, particular tagged entities are shortened, e.g., the prefixes or suffixes of antibody-related terms, as shown in the third row. For example, the species mentions “flag”, “goat” and “mouse” in “anti-**flag** antibody”, “**goat** anti-Rad53 polyclonal antibody” and “anti-HA9 **mouse** monoclonal antibody” are filtered.

**Table 4:** Types of species mentions extracted by two Inferring types.

Species type	Inferring type	Number of species mentions	Ratio
Genus name	Guaranteed-inference	2,824	8.06%
Cell line	Guaranteed-inference	715	2.04%
Sub-type	Co-occurrence inference	422	1.20%
General species name	-	31,093	88.70%
Total	-	35,054	100.00%

The total frequency of inference targets among species mentions is 11.3%.

**Table 5:** High-false-positive set.

Filter set	Terms
High-false-positive terms	3a (215167), t7 (10760), cat (9685), ass (9793), j1 (1829)
Experiment terms	yeast two hybrid (4932), yeast 2 hybrid (4932)
Antibody	anti-, antibodies, polyclonal, igg, serum, monoclonal, antibody

There are 5,933,419 Entrez Gene Ids belonging to more than 6,000 species. Genes are ambiguous among the other genes of the same species and among orthologs. Therefore, we developed a rule-based species assignation strategy to reduce orthologous gene ambiguity.

After the species is extracted, each gene entity is assigned the suitable tax_id. Based on Wang et al. [8], we defined several species indicators of tax_id assignment for gene mentions. Details are shown in Table 6. Boldface terms represent species names, and underlined words are gene mentions.

**Table 6:** Species indicators of species assignation strategy which are adapted from Wang et al. [8]

Species indicators	Description	Example
Identifier	The gene is assigned a species when the gene is extracted by gene identifier extraction.	A bar for Cat8 is not included, as only one gene (**YOR019W**, factor change 2.1) has a Cat8 site alone.

Prefix	The first lowercase letter of the gene name is an abbreviation of its species.	**h**ZIP 2 (human gene)

Previous indicator	The tax_id is assigned to a gene entity if the species entity appears in front of the gene entity.	**Drosophila**HCF **zebraflsh**Ltk

Forward indicator	The tax_id is assigned to a gene entity if the species entity is in front of the gene entity in the same sentence. The nearest species is used for assignment.	Conversely, ISWI is a unique and essential gene in **Drosophila**, highlighting a possible divergent role for ISWI in flies and a distinct mechanism of interaction with the Sin3A/Rpd3 complex in higher eukaryotes.

Backward indicator	The tax_id is assigned to a gene entity if the species entity precedes the gene entity in the same sentence. The nearest species is used for assignment.	We identify the shady gene as encoding a cell signaling receptor, leukocyte tyrosine kinase (Ltk), that has recently been associated with **human** auto-immune disease.

Majority indicator	The most frequently mentioned tax_id is assigned to the gene entity if it cannot be assigned by previous rules.	The most frequent mention of PMCID: 2880583 is tax_id is 9606 (Human) as shown in Table 7.

Table 7 shows an example using the frequency of species mentions from PMCID: 2880583. The tax_id:9606 (Human) is used most frequently in this article, and there is 1 instance of “293 cell”, 34 instances of “a549 cell” and 6 instances of “human”. If there are no other species mentions in the article, all gene mentions are assigned by tax_id: 9606 (human).

**Table 7:** An example of the frequency of species mentions by PMCID: 2880583.

PMCID	Species and cell name	tax id	Num
2880583	293(1), a549(34), human(6)	9606	41
	bovine(1)	9913	1
	mice(1), murine(1)	10090	2
	rabbit(1)	9986	1
	sendai virus(2)	11191	2

### Species-specific gene normalization module

After species assignation, the proposed SGN module, based on previous work [23], measures the inference scores of candidate Entrez Gene Ids in articles. The previous study applied an inference network model to the GN task, but it did not handle orthologous gene ambiguity. We applied the inference network model to collect the exact match and partial match between tagged entities from articles and gene name entities from the gene dictionary. The inference by exact match is named entity inference, and the inference by partial match is named bag of word inference. Exact match means that tagged entity and gene name entity must be the same term, and partial match means that at least one word in the bags of words of a tagged entity and of a gene name entity must be the same word. The model applied entities and bag of words to the same estimation. The original model gave equal weight to entities and bags of words, thus could not distinguish the relative importance of entities and words from the bags of words. This design disadvantaged the GN performance. We reorganized the previous design by splitting the inference network model into two inference estimations.

**Figure 4:**
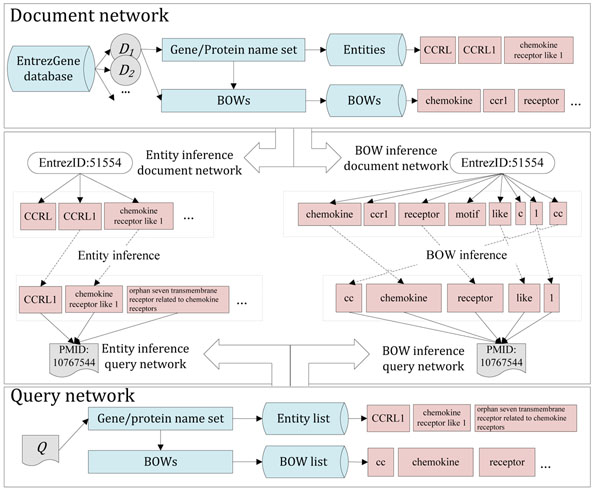
Example of species-specific gene normalization by two inference estimations including entity and BOW inference.

In SGN, entity inference and bag-of-words inference are used to measure confidence scores. The gene name entities are divided into two lists, an entity list and a bag-of-words list. The entity list stores the output of the GNR module, and the bag of words list contains all bags of words from the entity list. Each record of these two lists is used to obtain a candidate Entrez Gene Id (*Cid*). Before inference estimations, the *Cid* is filtered by the intersection filtering method described below. The two inference estimations apply a TF-IDF-based inference network to determine the possible Entrez Gene Ids for each article. Consider the inference example in Figure 4, Entrez Gene Id: 51554 includes entities “CCRL”, “CCRL1”, “chemokine receptor like 1” and “orphan seven-transmembrane receptor”, etc. PMID: 10767544 consists of entities “CCRL1”, “chemokine receptor like 1” and “orphan seven transmembrane receptor related to chemokine receptors”, etc. Those entities are used to construct entity inference and bag-of-word inference estimations. The entity inference is constructed by the exact match between gene name entities from Entrez Gene Id: 51554 and tagged entities from PMID: 10767544. There are two exact matches in entity inference, e.g. “CCRL1” and “chemokine receptor like 1”. Then, the bag-of-word inference is constructed by partial match between bags of words of gene name entities from Entrez Gene Id: 51554 and tagged entities from PMID: 10767544, such as “chemokine”, “receptor” and “like”.

For disambiguating confused *Cid*s and decreasing the computation cost, we discard many irrelevant *Cid*s by our intersection filtering method. All pairs of *Cid*s are compared: all terms extracted by exact and partial match are considered in each *Cid*. A *Cid* is removed when its term list is a subset of the term list of another *Cid.*

## Results

Our system was run on the evaluation data of the BioCreative III GN [24] task training and test corpora, as shown in Table 8. The test data were unknown to our system until the official runs were executed. The training set included two sets of annotated full-length articles. The first set had been fully annotated by a group of trained and experienced curators, who had been invited from various model organism databases. The second set was partially annotated: only the most important genes had been annotated by human indexers at the National Library of Medicine. The test set included 507 full text articles from various BMC and PLoS journals. To understand the differences in the results between teams, organizers selected the 50 articles that presented the most difficult and varied results to evaluate the submissions of the teams. The “gold standard” of the 50 selected articles was annotated manually, and the “silver standard” of 507 articles including the 50 “gold standard” articles was generated automatically by an Expectation Maximization (EM) algorithm using the best submissions of all teams. The numbers of gene IDs common to the shared 50 gold- and silver-standard articles is just 528. The annotated gene identifiers of the gold standard are very dissimilar to silver standard.

**Table 8:** Statistics of annotations of full-text articles

	Training (fully annotated)	Training (partially annotated)	Test (gold standard)	Test (silver standard)	
	
				507	50
Total number of full-text articles	32	525	50	507	50
Total gene IDs of each annotation	607	770	1669	9378	1828
Avg. gene IDs of each annotation	18.97	1.467	33.32	18.50	36.56

Carrol et al. [25] proposed a new metric, threshold average precision (TAP-k), for measuring retrieval efficacy of GN task performance. In short, TAP-k is the mean average precision with a variable cutoff and a terminal cutoff penalty. Evaluations using the gold and silver standard annotations are shown in Table 9; note that the set of 50 gold standard articles is a part of the 507 silver standard articles. We obtained the highest TAP-k scores on the gold standard: 0.3297 (k=5), 0.3538 (k=10), and 0.3535 (k=20). In silver-standard-507 evaluation, our TAP-k scores are 0.4591 for k=5, 10, and 20. Our system was ranked 2^nd^, 2^nd^, and 3^rd^ in terms of TAP-5, TAP-10, and TAP-20 respectively on the 507 full-text test articles.

**Table 9:** Performance on the gene normalization task by the top 4 performing teams in the BioCreative III competition

Teams		Gold standard (50 selected articles)			Silver standard (50 selected articles)			Silver standard (All 507 articles)		
	
		TAP-5	TAP-10	TAP-20	TAP-5	TAP-10	TAP-20	TAP-5	TAP-10	TAP-20
Kuo et al. (Team 74)	1st run	0.2137	0.2509	0.2509	0.3820	0.3820	0.3820	0.4873	0.4873	0.4873
	2nd run	0.2083	0.2480	0.2480	0.3855	0.3855	0.3855	0.4871	0.4871	0.4871
	3rd run	0.2099	0.2495	0.2495	**0.3890**	0.3890	0.3890	**0.4916**	**0.4916**	**0.4916**

Our method (Team 83)	1st run	0.3254	**0.3538**	**0.3535**	0.3567	0.3600	0.3600	0.4591	0.4591	0.4591
	2nd run	0.3216	0.3435	0.3435	0.3291	0.3291	0.3291	0.4323	0.4323	0.4323
	3rd run	**0.3297**	0.3514	0.3514	0.3382	0.3382	0.3382	0.4327	0.4327	0.4327

Liu et al. (Team 98)	1st run	0.2835	0.3012	0.3103	0.3343	0.3535	0.3629	0.3818	0.3899	0.3875
	2nd run	0.2909	0.3079	0.3087	0.3354	0.3543	0.3634	0.3790	0.3878	0.3868
	3rd run	0.3013	0.3183	0.3303	0.3710	**0.4116**	**0.4672**	0.4086	0.4511	0.4648

Lai et al. (Team 101)	1st run	0.1896	0.2288	0.2385	0.3590	0.3859	0.3859	0.4289	0.4289	0.4289
	2nd run	0.1672	0.2150	0.2418	0.3239	0.3945	0.4132	0.4294	0.4408	0.4408
	3rd run	0.1812	0.2141	0.2425	0.3258	0.4109	0.4109	0.4536	0.4536	0.4536

The annotation of the silver standard depends on the submissions of all teams. Because the best submission might be dissimilar to other teams’ submissions, its relative performance on the silver standard might suffer. Nevertheless, the two best runs with the gold standard from our submission are still among the ones with the highest TAP-k score with the silver standard. In addition, the four teams (numbers 83, 74, 98 and 101) that performed best on the gold standard consistently remained in the top tier in silver-standard evaluations. It is evident that relative rankings tended to be preserved in this comparison. Evaluation with the silver-standard annotation proves that the automatic annotation works [24].

In addition, we calculated precision, recall and F-measure to evaluate the accuracy and coverage of our result (Team 83). We obtained an F-measure of 46.56% with the gold standard, 46.90% with the silver standard with 50 articles and 55.09% with the silver standard with 507 articles (Table 10).

**Table 10:** Performance statistics evaluated by TAP-K and F-measure on test data and training data sets.

Corpus	Data set	TAP-5	TAP-10	TAP-20	Precision	Recall	F-measure
Test data (1st run)	50 (gold standard)	0.3254	**0.3538**	**0.3535**	53.85%	39.44%	45.53%
Test data (2nd run)	50 (gold standard)	0.3216	0.3435	0.3435	55.54%	39.07%	45.87%
Test data (3rd run)	50 (gold standard)	**0.3297**	0.3514	0.3514	**56.23%**	**39.72%**	**46.56%**

Test data (1st run)	50 (silver standard)	**0.3567**	**0.3600**	**0.3600**	58.94%	**38.95%**	**46.90%**
Test data (2nd run)	50 (silver standard)	0.3291	0.3291	0.3291	58.60%	37.64%	45.84%
Test data (3rd run)	50 (silver standard)	0.3382	0.3382	0.3382	**59.46%**	38.35%	46.62%

Test data (1st run)	507(silver standard)	**0.4591**	**0.4591**	**0.4591**	71.79%	**44.69%**	**55.09%**
Test data (2nd run)	507(silver standard)	0.4323	0.4323	0.4323	72.08%	42.70%	53.64%
Test data (3rd run)	507(silver standard)	0.4327	0.4327	0.4327	**72.41%**	42.82%	53.82%

Training data	32 (gold standard)	0.4703	0.4969	0.4969	63.82%	67.71%	65.70%

To better understand the contribution of each component in the GN method, we sequentially ran the system over the test data without each component of each module. Table 11 shows how each component contributed to GN performance. The first row shows the TAP-k (k=5, 10, 20) score when all components were used. The other rows show the performance when one of the components was missing. The values in parentheses are the decrement of the component removals.

**Table 11:** Contribution of each component of modules

Remove component	TAP-5	TAP-10	TAP-20
None	0.3254	0.3538	0.3535
**GNR module**			
Post-processing of AIIA-GMT [22]	0.2653(-18.47%)	0.3063(-13.43%)	0.3063(-13.35%)
Distillation strategy	0.3094(-4.92%)	0.3355(-5.17%)	0.3355(-5.09%)
Identifier extraction	0.2706(-16.84%)	0.2888(-18.37%)	0.2888(-18.30%)
**SA module**			
Two robust inference strategies	0.3014(-7.38%)	0.3319(-6.19%)	0.3319(-6.11%)
Identifier	0.2538(-22.00%)	0.2538(-28.26%)	0.2644(-25.21%)
Prefix	0.3119(-4.15%)	0.3383(-4.38%)	0.3383(-4.30%)
Previous indicator	0.3099(-4.76%)	0.3361(-5.00%)	0.3361(-4.92%)
Forward indicator	0.2846(-12.54%)	0.3056(-13.62%)	0.3056(-13.55%)
Backward indicator	0.3056(-6.08%)	0.3330(-5.88%)	0.3330(-5.80%)
Majority indicator	0.2280(-29.93%)	0.2280(-35.56%)	0.2280(-35.50%)
**SGN module**			
Intersection filtering method	0.3121(-4.09%)	0.3411(-3.59%)	0.3411(-3.51%)
Inference network	0.2852(-12.35%)	0.3039(-14.10%)	0.3039(-14.03%)

## Discussion

In the following section, we first discuss the impact of the GNR module in the context of three extension components of the gene mention recognition system (AIIA-GMT). The impact of two robust referring strategies, the filtering processing and five assignation rules (because identifier extraction is repeated in the GNR module, we do not perform the same experiment again) of SA module are described. Finally, we analyze the performance of the SGN module by the intersection filtering method and inference network.

First, we found that the post-processing step is a useful component of the GNR module. If the output of AIIA-GMT were not followed by post-processing, performance would clearly decrease. The most effective component is identifier extraction; several popular and high-performance tagging systems, such as AIIA-GMT [22], ABNER [26], and GENIA Tagger [27], usually cannot recognize gene identifiers well.

The three rows under the SA module (Table 11) show the performance when one of the two robust inference strategies, the filtering processing and six species assignation rules, was unused. The disambiguation of species mentions is critical, which is why we designed two robust referring strategies to handle it. Using only one of the two robust referring strategies caused a decline in the performance of the species assignation rules and an approximately 6%-7% decrease in TAP-k scores. The effect of the two robust inference strategies is not obvious; the ratio of species mentions that are unguaranteed species names (i.e., genus and cell line) and sub-type is just 11.3% (Table 4). The filtering of false-positive set is very useful. The next most helpful indicators of species assignation are “Forward indicator” and “Majority indicator”. Especially for “Majority indicator”, the detection of the species covered in the article is an important issue [8,10]. Majority indicator is most popular. Furthermore, the precise extraction of species mentions leads to good performance of the Majority indicator.

Finally, the SGN module includes two major parts: intersection filtering and inference estimation. The contribution of the intersection filtering method is to enhance computing speed, and thus the effect of this method on performance is not remarkable. To evaluate the contribution of the inference network model of two estimations, we replaced this model by a simple vector space model. The performance decreased substantially.

We evaluated our method using the full-text articles provided in the BioCreative III competition. Our best result had TAP-k scores of 0.3297 (k=5), 0.3538 (k=10), and 0.3535 (k=20) under the gold-standard evaluation.

Our method approaches the challenges of GN as a series of tasks, with several issues handled by respective modules. The disadvantageous designs of each module may lead to a decline in overall performance. The major goal of this work is to present the architecture of our method in a clear way and analyze the effective components, which we do through systematic removals. This analysis can help in the redesigning of each segment to create a better system.

## Conclusions

The GN task of BioCreative III was more difficult than previous GN tasks, and the chief reason is orthologous gene ambiguity. In this study, we focused on the issue of gene normalization in species assignation and developed an integrated method for mapping a biomedical entity to the correct Entrez Gene Id. To obtain good performance, we focused on ameliorating the effects of gene mention variation, orthologous gene ambiguity and intra-species gene ambiguity. The integrated method consists of three modules, GNR, SA and SGN, which function serially to handle these three issues. We participated in the GN task of the BioCreative III competition by adopting an integrated method based on our previous work to handle intra-species gene ambiguity. Results demonstrated that our method worked well, ranking at the top level of performance among all teams. Our proposed method makes sufficient use of gene/species information in context and of a thesaurus of gene/species.

Nonetheless, the current, state-of-the-art performance on the GN task is not good enough. The mining of full-text articles and cross-species normalization are big challenges for GN. To improve future performance, the contexts of articles will be used, e.g., chromosomal locations, families, functions.

## Competing interests

The authors declare that they have no competing interests.

## References

[B1] Alex B Grover C Haddow B Kabadjov M Klein E Matthews M Roebuck S Tobin R Wang X Assisted curation: does text mining really help? Pac Symp Biocomput 2008 556 567 18229715

[B2] Hakenberg J Plake C Leaman R Schroeder M Gonzalez G Inter-species normalization of gene mentions with GNAT Bioinformatics 2008 24 ECCB i126 i132 18689813 10.1093/bioinformatics/btn299

[B3] Heinz JF Mevissen T Dach H Oster M Hofmann-Apitius M ProMiner: Recognition of Human Gene and Protein Names using regularly updated Dictionariesthe Second BioCreative Challenge Evaluation Workshop 2007 149 151

[B4] Hirschman L Colosimo M Morgan A Yeh A Overview of BioCreAtIvE task 1B: normalized gene lists BMC Bioinformatics 2005 6 Suppl 1 S11 10.1186/1471-2105-6-S1-S11 15960823 PMC1869004

[B5] Leitner F Mardis SA Krallinger M Cesareni G Hirschman LA Valencia A An Overview of BioCreative II.5 IEEE/ACM Transactions On Computational Biology And Bioinformatics 2010 7 3 385 399 20704011 10.1109/tcbb.2010.61

[B6] Morgan AA Lu Z Wang X Cohen AM Fluck J Ruch P Divoli A Fundel K Leaman R Hakenberg J Overview of BioCreative II gene normalization Genome Biology 2008 9 Suppl 2 S3 10.1186/gb-2008-9-s2-s3 18834494 PMC2559987

[B7] Wermter J Tomanek K Hahn U High-Performance Gene Name Normalization with GENO Bioinformatics 2009 10.1093/bioinformatics/btp071 19188193

[B8] Wang X Tsujii Ji Ananiadou S Disambiguating the Species of Biomedical Named Entities using Natural Language Parsers Bioinformatics 2010 26 5 661 667 10.1093/bioinformatics/btq002 20053840 PMC2828111

[B9] Gerner M Nenadic G Bergman CM LINNAEUS: A species name identification system for biomedical literature BMC Bioinformatics 2010 11 85 10.1186/1471-2105-11-85 PMC2836304 20149233

[B10] Kappeler T Kaljurand K Rinaldi F TX Task:Automatic Detection of Focus Organisms in Biomedical Publications Proceedings of the Workshop on BioNLP: 2009 2009 80 88

[B11] Klinger R Kolarik C Fluck J Hofmann-Apitius M Friedrich CM Detection of IUPAC and IUPAC-like chemical names Bioinformatics 2008 24 ISMB2008 i268 i276 18586724 10.1093/bioinformatics/btn181 PMC2718657

[B12] Corbett P Batchelor C Teufel S Annotation of Chemical Named Entities BioNLP 2007: Biological, translational, and clinical language processing 2007 57 64

[B13] Hakenberg J Plake C Royer L Strobelt H Leser U Schroeder M Gene mention normalization and interaction extraction with context models and sentence motifs Genome Biology 2008 9 Suppl 2 S14 10.1186/gb-2008-9-s2-s14 18834492 PMC2559985

[B14] Hakenberg J Leaman R Vo NH Jonnalagadda S Miller RSC Tari L Baral C Gonzalez G Efficient Extraction of Protein-Protein Interactions from Full-Text Articles IEEE/ACM Transactions On Computational Biology And Bioinformatics 2010 7 3 481 494 20498514 10.1109/TCBB.2010.51

[B15] Chen Y Liu F Manderick B BioLMiner System: Interaction Normalization Task and Interaction Pair Task in the BioCreative II.5 Challenge IEEE/ACM Transactions On Computational Biology And Bioinformatics 2010 7 3 428 441 20671315 10.1109/TCBB.2010.47

[B16] Verspoor K Roeder C Johnson HL Cohen KB Jr. W AB Hunter LE Exploring Species-Based Strategies for Gene Normalization IEEE/ACM Transactions On Computational Biology And Bioinformatics 2010 7 3 462 471 20671318 10.1109/TCBB.2010.48 PMC2929766

[B17] Saetre R Yoshida K Miwa M Matsuzaki T Kano Y Tsujii Ji Extracting Protein Interactions from Text with the Unified AkaneRE Event Extraction System IEEE/ACM Transactions On Computational Biology And Bioinformatics 2010 7 3 442 453 20671316 10.1109/TCBB.2010.46

[B18] Dai HJ Lai PT Tsai RTH Multistage Gene Normalization and SVM-Based Ranking for Protein Interactor Extraction in Full-Text Articles IEEE/ACM Transactions On Computational Biology And Bioinformatics 2010 7 3 412 420 20479501 10.1109/TCBB.2010.45

[B19] Maglott D Ostell J Pruitt KD Tatusova T Entrez Gene: gene-centered information at NCBI Nucleic acids research 2006 00 Database issue D1 D6 10.1093/nar/gki031 PMC539985 15608257

[B20] Hirschman L Morgan AA Yeh AS Rutabaga by any other name: extracting biological names J of Biomedical Informatics 2002 35 4 247 259 10.1016/S1532-0464(03)00014-5 12755519

[B21] Tuason O Chen L Liu H Blake JA Friedman C Biological Nomenclatures: A Source of Lexical Knowledge and Ambiguity Proc Pacific Symp on Biocomputing 2004 238 249 10.1142/9789812704856_0023 14992507

[B22] Hsu CN Chang YM Kuo CJ Lin YS Huang HS Chung IF Integrating High Dimensional Bi-directional Parsing Models for Gene Mention Tagging Bioinformatics 2008 24 ISMB2008 i286 i294 18586726 10.1093/bioinformatics/btn183 PMC2718659

[B23] Wei CH Huang IC Hsu YY Kao HY Normalizing Biomedical Name Entities by Similarity-Based Inference Network and De-ambiguity Mining Ninth IEEE International Conference on Bioinformatics and Bioengineering Workshop: Semantic Biomedical Computing: 2009; Taichung, Taiwan 2009 461 466

[B24] Lu Z Wilbur WJ Overview of BioCreative III Gene Normalization BioCreative III Workshop 2010 Maryland,Bethesda

[B25] Carroll HD Kann MG Sheetlin SL Spouge JL Threshold Average Precision (TAP-k): A Measure of Retrieval Designed for Bioinformatics Bioinformatics 2010 26 14 1708 1713 10.1093/bioinformatics/btq270 20505002 PMC2894514

[B26] Settles B ABNER: an open source tool for automatically tagging genes, proteins and other entity names in text Bioinformatics 2005 21 14 3191 3192 10.1093/bioinformatics/bti475 15860559

[B27] Kim JD Ohta T Tateisi Y Tsujii J GENIA corpus-a semantically annotated corpus for bio-textmining Bioinformatics 2003 19 Suppl. 1 i180 i182 12855455 10.1093/bioinformatics/btg1023

